# Supplementary material for: The first juvenile specimens of Plateosaurus engelhardti from Frick, Switzerland: isolated neural arches and their implications for developmental plasticity in a basal sauropodomorph
Source: PeerJ. 2014 Jul 3;2:e458. doi: 10.7717/peerj.458 (PMC4103078; doi:10.7717/peerj.458)
Supplement: Table S1 — Zygapophyses lengths in mm of the juvenile specimens MSF 11.3., MSF 5B, MSF 23 and SMNS 13200 with their respective position in the vertebral column. As noted in the description some positions can be recognized twice. The zygapophyses lengths in parentheses belong to the longer neural arch of those to be found twice in the sample. The specimen numbers of the 11.3. specimens is listed in Table 1. D3 (MSF 11.3.376/77.1 mm and MSF 11.3.360/86.7 mm). D5 (MSF 11.3.167/94.2 mm and MSF 11.3.067/101.1 mm). D6 (MSF 11.3.095/108.5 mm and MSF 11.3.107/109.2 mm). D10/D11 (MSF 11.3.241/110.8 mm and MSF 11.3.303/121.6 mm). Gaps are left, where data is missing due to preservation or the accessibility is was not given. [file peerj-02-458-s001.docx]

| Position in vertebral column | MSF 11.3.  (mm) | MSF 5B (mm) | MSF 23 (mm) | SMNS 13200 (mm) |
| --- | --- | --- | --- | --- |
| C1 |  |  |  |  |
| C2 (axis) | 77.4 | 86.7 | 76.2 |  |
| C3 | 117.7 | 149.7 | 102.0 | 146.8 |
| C4 | 142.5 | 160.0 | 144.5 | 171.0 |
| C5 |  | 159.0 | 157.0 | 172.0 |
| C6 | 129.7 | 152.0 | 155.0 | 151.5 |
| C7 |  | 155.0 |  | 177.0 |
| C8 |  | 130.1 | 126.5 | 175.0 |
| C9 |  | 135.8 |  | 134.0 |
| C10 | 109.9 | 139.9 | 90.9 | 130.0 |
| D1 |  | 106.7 | 98.4 | 121.7 |
| D2 |  | 106.1 | 97.2 | 113.0 |
| D3 | 77.1 (86.7) | 101.9 | 99.0 | 110.0 |
| D4 | 98.7 | 112.7 |  | 114.0 |
| D5 | 94.2 (101.1) | 114.0 |  |  |
| D6 | 108.5 (109.2) |  | 118.4 | 135.0 |
| D7 | 106.6 |  |  | 140.0 |
| D8 |  |  | 120.8 |  |
| D9 |  |  | 131.1 | 122.0 |
| D10 (/D11) | 110.8 (121.6) |  | 135.1 | 140.0 |
| D11 |  |  | 135.0 | 142.0 |
| D12 |  |  | 116.0 | 134.2 |
| D13 |  |  |  | 143.5 |
| D14 |  |  |  | 133.0 |
| D15 |  |  |  |  |
