# Supplementary material for: The first juvenile specimens of Plateosaurus engelhardti from Frick, Switzerland: isolated neural arches and their implications for developmental plasticity in a basal sauropodomorph
Source: PeerJ. 2014 Jul 3;2:e458. doi: 10.7717/peerj.458 (PMC4103078; doi:10.7717/peerj.458)
Supplement: Table S2 — shows the calculated ratio in percent between the zygapophyses length and the femur length of the referred specimens. The ratios within on specimen and compared to each other specimen follow a determined scheme of zygapohyseal length explaining the wide range between 12.5 and 28.3%. With the help of the ratios we were able to define a new proxy for the determination of a range of femur lengths for the juvenile MSF 11.3. specimens. [file peerj-02-458-s002.docx]

| Position in vertebral column | MSF 5B (%) | MSF 23 (%) | SMNS 13200 (%) |
| --- | --- | --- | --- |
| C1 |  |  |  |
| C2 (axis) | 15.3 | 12.5 |  |
| C3 | 26.5 | 16.7 | 21.4 |
| C4 | 28.3 | 23.7 | 25.0 |
| C5 | 28.1 | 25.7 | 25.1 |
| C6 | 26.9 | 25.4 | 22.1 |
| C7 | 27.4 |  | 25.8 |
| C8 | 23.0 | 20.7 | 25.5 |
| C9 | 24.0 |  | 19.6 |
| C10 | 24.8 | 14.9 | 19.0 |
| D1 | 18.9 | 16.1 | 17.8 |
| D2 | 18.8 | 15.9 | 16.5 |
| D3 | 18.0 | 16.2 | 16.1 |
| D4 | 19.9 |  | 16.6 |
| D5 | 20.2 |  |  |
| D6 |  | 19.4 | 19.7 |
| D7 |  |  | 20.4 |
| D8 |  | 19.8 |  |
| D9 |  | 21.5 | 17.8 |
| D10 |  | 22.1 | 20.4 |
| D11 |  | 22.1 | 20.7 |
| D12 |  | 19.0 | 19.6 |
| D13 |  |  | 20.9 |
| D14 |  |  | 19.4 |
| D15 |  |  |  |
