# Supplementary figures and images for: The first juvenile specimens of Plateosaurus engelhardti from Frick, Switzerland: isolated neural arches and their implications for developmental plasticity in a basal sauropodomorph
Source: PeerJ. 2014 Jul 3;2:e458. doi: 10.7717/peerj.458 (PMC4103078; doi:10.7717/peerj.458)

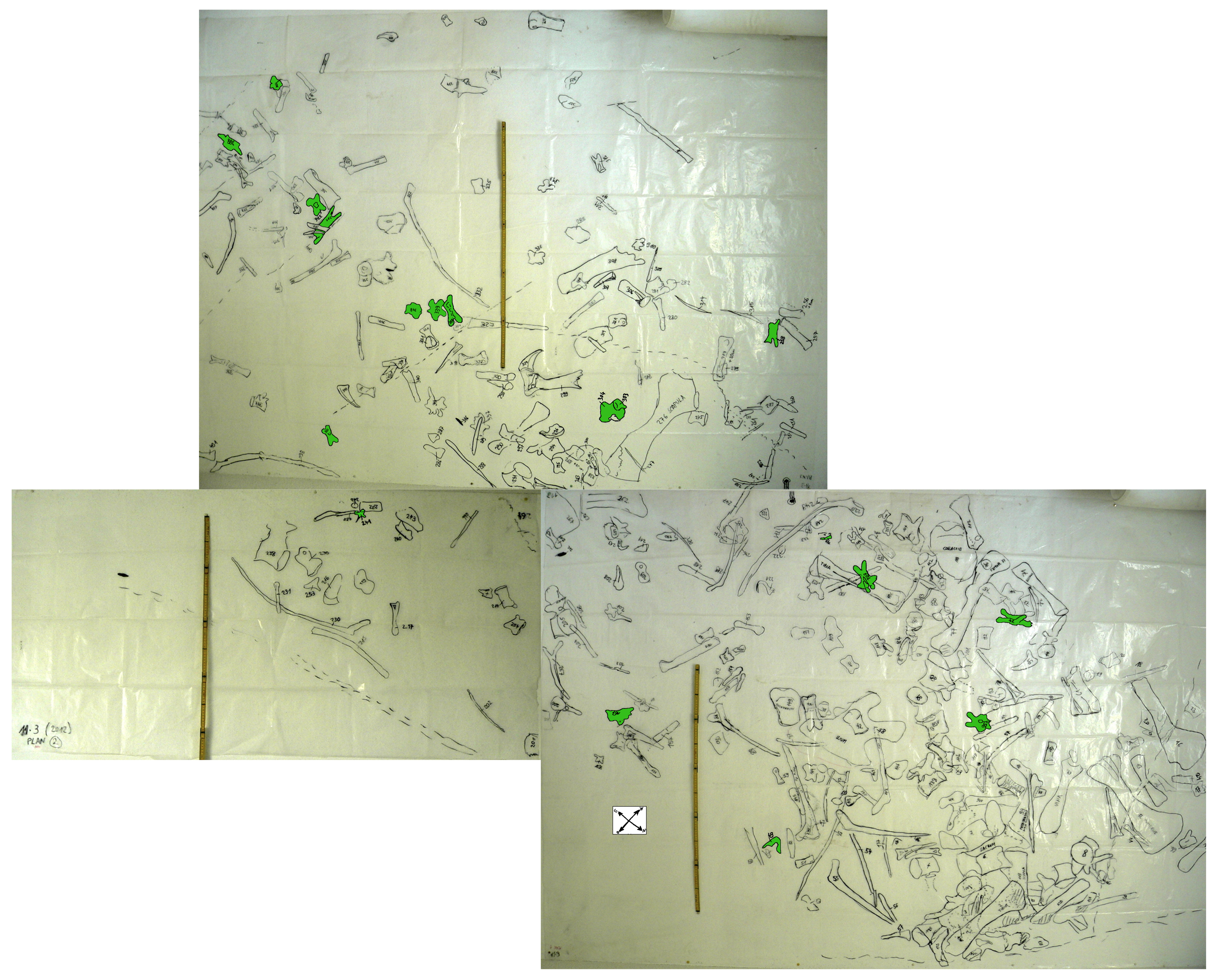

Supplement: Figure S1 — The foil plan shows the isolated neural arches found in bone field 11.3. The neural arches are colored in green. The plan clearly shows that the neural arches were distributed over the whole area with no recognizable connection to each other and no centra lying next to them. The yardstick measures 1 m. [file peerj-02-458-s003.png]

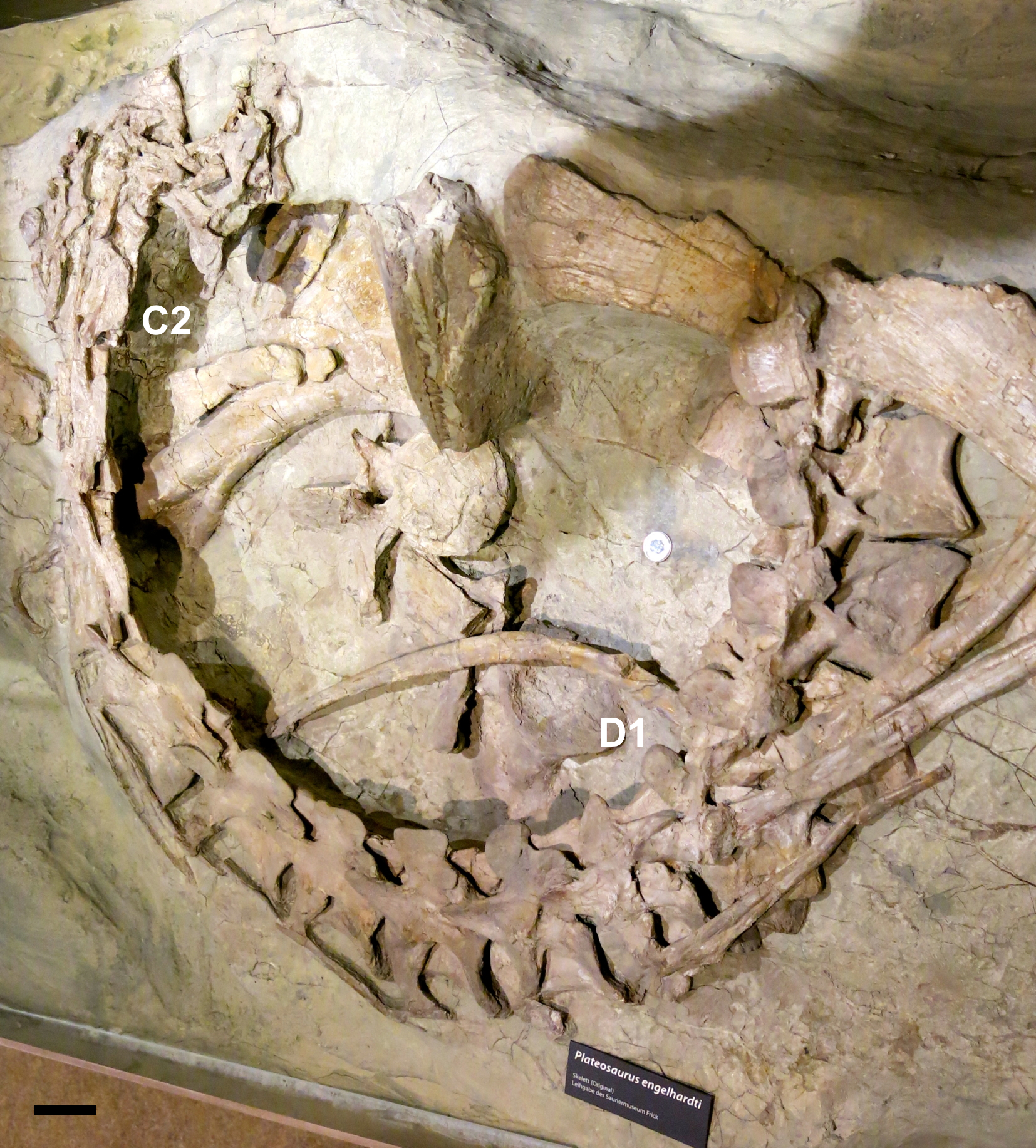

Supplement: Figure S2 — Specimen MSF 5B reveals a complete articulated cervical series from vertebrae C2 to C10 and articulated dorsal vertebrae from D1 to D5. MSF 5B. All zygapophyseal lengths were available for measurements. MSF 5B being an osteologically mature specimen of Plateosaurus engelhardti shows completely closed neurocentral sutures with all morphological characters being well developed. Scale bar measures 5 cm. [file peerj-02-458-s004.png]

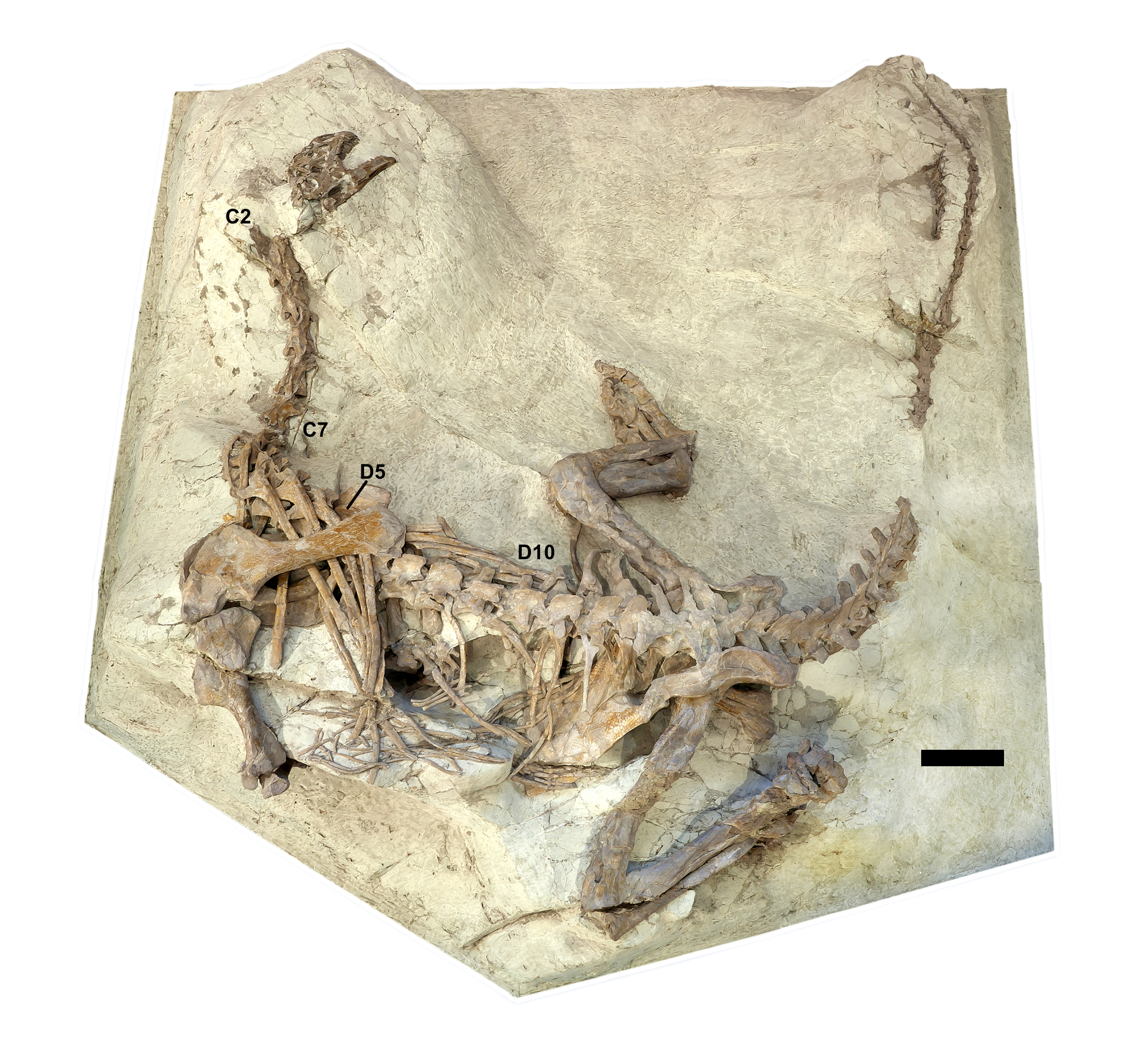

Supplement: Figure S3 — Specimen MSF 23 is a nearly complete and in most parts articulated P. engelhardti. The cervical vertebrae series is complete from C2 to C10. The dorsal series is complete from D1 to D15. The vertebrae of this specimen are heavily deformed, especially in the posterior dorsal series making measurements difficult. This specimen shows completely closed neurocentral sutures and all morphological characters are well developed. Scale bar measures 20 cm. [file peerj-02-458-s005.png]

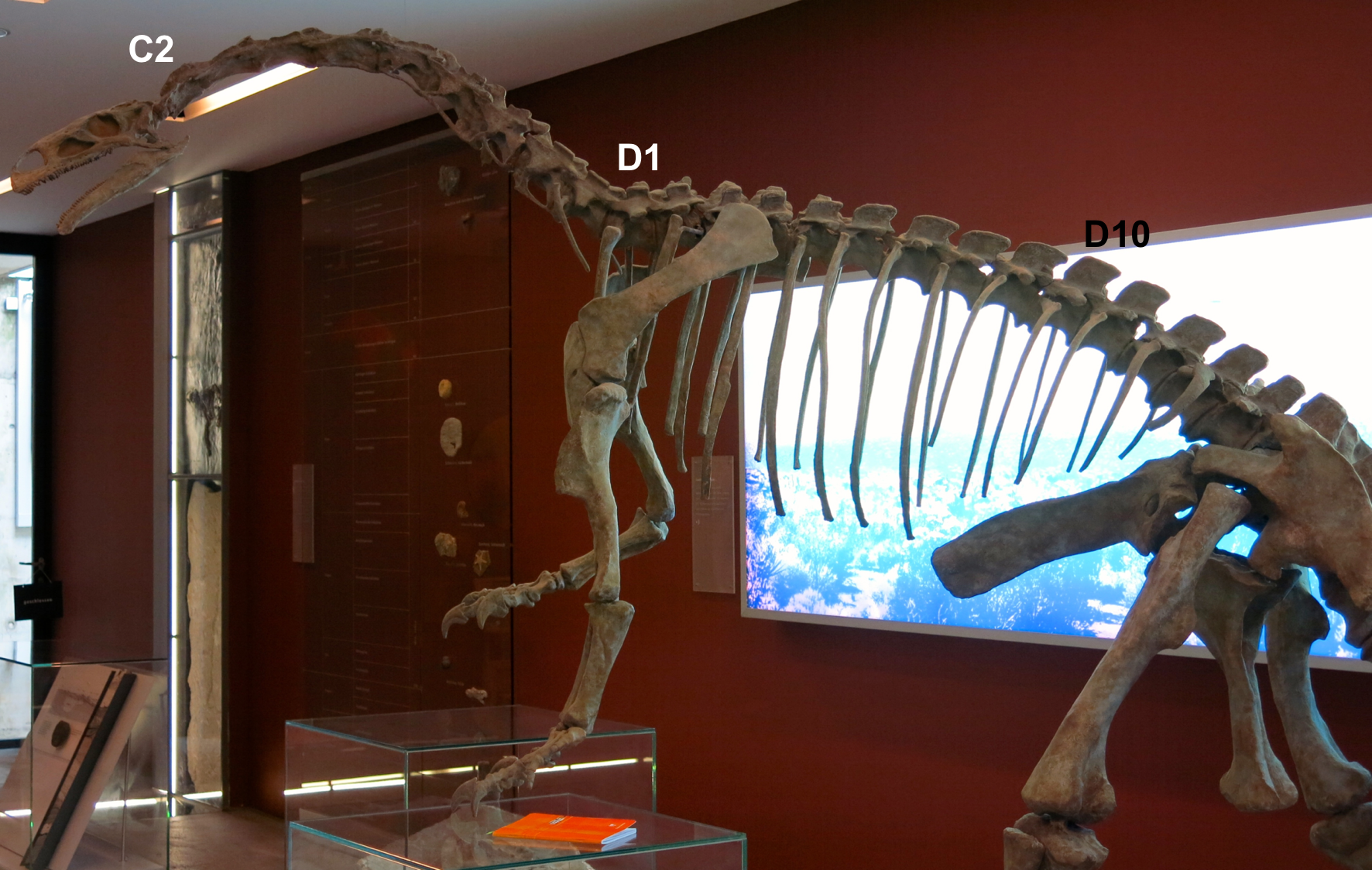

Supplement: Figure S4 — A complete mounted skeleton cast of SMNS 13200 from Trossingen, Germany. The cervical as well as the dorsal vertebrae series is well preserved. All neurocentral sutures are completely closed and all morphological characters are well developed. For scaling: the left femur length of specimen SMNS 13200 measures 68.5 cm. [file peerj-02-458-s006.png]
